# TNF-α-Inhibition Improves the Biocompatibility of Porous Polyethylene Implants* In Vivo*

**DOI:** 10.1007/s13770-020-00325-w

**Published:** 2021-01-30

**Authors:** Timon Hussain, Donata Gellrich, Svenja Siemer, Christoph A. Reichel, Jonas Eckrich, Dimo Dietrich, Shirley K. Knauer, Roland H. Stauber, Sebastian Strieth

**Affiliations:** 1 Walter Brendel Centre of Experimental Medicine, Ludwig-Maximilians-University Munich, Marchioninistr. 27, 81377 Munich, Germany; 2 Department of Otorhinolaryngology, Head and Neck Surgery, University of Duisburg-Essen, Hufelandstr. 55, 45147 Essen, Germany; 3 Department of Otorhinolaryngology, Head and Neck Surgery, Ludwig-Maximilians-University Munich, Marchioninistr. 27, 81377 Munich, Germany; 4 Department of Otorhinolaryngology, Head and Neck Surgery, Johannes Gutenberg University Mainz, Langenbeckstr. 1, 55131 Mainz, Germany; 5 Department of Otorhinolaryngology, Head and Neck Surgery, University of Bonn, Venusberg Campus 1, 53127 Bonn, Germany; 6 Department of Molecular Biology, Centre for Nanointegration (CENIDE), University Duisburg-Essen, Universitätsstraße 5, 45117 Essen, Germany

**Keywords:** Porous polyethylene, Etanercept, Implant integration, Fluorescence microscopy, ECM

## Abstract

**Background:**

To improve the biocompatibility of porous polyethylene (PPE) implants and expand their application range for reconstructive surgery in poorly vascularized environments, implants were coated with tumor necrosis factor α (TNFα) inhibitor Etanercept. While approved for systemic application, local application of the drug is a novel experimental approach. Microvascular and mechanical integration as well as parameters of inflammation were analyzed *in vivo*.

**Methods:**

PPE implants were coated with Etanercept and extracellular matrix (ECM) components prior to implantation into dorsal skinfold chambers of C57BL/6 mice. Fluorescence microscopy analyses of angiogenesis and local inflammatory response were thrice performed *in vivo* over a period of 14 days to assess tissue integration and biocompatibility. Uncoated implants and ECM-coated implants served as controls.

**Results:**

TNFα inhibition with Etanercept led to a reduced local inflammatory response: leukocyte-endothelial cell adherence was significantly lowered compared to both control groups (*n* = 6/group) on days 3 and 14, where the lowest values were reached: 3573.88 leukocytes/mm-2 ± 880.16 (uncoated implants) vs. 3939.09 mm-2 ± 623.34 (Matrigel only) vs. 637.98 mm-2 + 176.85 (Matrigel and Etanercept). Implant-coating with Matrigel alone and Matrigel and Etanercept led to significantly higher vessel densities 7 and 14 days vs. 3 days after implantation and compared to uncoated implants. Mechanical implant integration as measured by dynamic breaking strength did not differ after 14 days.

**Conclusion:**

Our data show a reduced local inflammatory response to PPE implants after immunomodulatory coating with Etanercept *in vivo*, suggesting improved biocompatibility. Application of this tissue engineering approach is therefore warranted in models of a compromised host environment.

**Electronic supplementary material:**

The online version of this article (10.1007/s13770-020-00325-w) contains supplementary material, which is available to authorized users.

## Introduction

Porous polyethylene (PPE) implants are routinely used for various applications in plastic and reconstructive surgery. Reported functional and aesthetic results after surgical procedures such as rhinoplasty, orbital floor reconstruction, auricular reconstruction, or facial augmentation are favorable [1–4], with low complication rates. Nevertheless, challenges can arise under circumstances where biointegration is compromised, i.e. during revision surgery or after radiation therapy. Here, limited tissue vascularization can delay vessel ingrowth and lead to an excessive inflammatory response by the host—both are key obstacles for successful and sustainable biointegration [5, 6]. Previous experimental approaches to further improve the biocompatibility of Medpor^®^ PPE implants include coating implants with extracellular matrix components and vascular endothelial growth factor [7]. Hereby, the initial inflammatory response by the host was significantly reduced, as measured by leukocyte-endothelial cell interactions. Applying components of the plasminogen activation cycle, specifically urokinase-type plasminogen activator, tissue plasminogen activator, plasminogen activator inhibitor-1, or vitronectin accelerated implant vascularization and improved mechanical integration [8, 9]. Other approaches were based on a vitalization of implants by pre-operatively coating PPE scaffolds with vital fibroblasts, or chondrocytes [10, 11]. The latter also led to an improvement in vascularization.

In anticipation of a potentially rapid translation to clinical application, in this study, PPE implants were coated with the tumor necrosis factor α (TNFα) inhibitor Etanercept prior to implantation. Etanercept is an approved drug for the treatment of rheumatoid arthritis [12], which functions as a decoy receptor that binds to TNFα and β. Etanercept is a drug which is commonly applied systemically but conjugation of Etanercept to synthetic microspheres has been shown to be feasible and achieved lasting release effects *in vitro* [13]. At this point, Etanercept is not an established therapeutic agent for tissue engineering, however, its effects may prove beneficial in improving implant biocompatibility of PPE implants. TNFα activates a broad spectrum of intracellular signaling mechanisms and is known to be one of the central stimuli in the inflammatory cascade, as shown in other biomaterial studies, primarily for joint implants [14, 15]. For PPE implants,* in vitro* studies recently demonstrated an association between increased TNFα expression and macrophage proliferation [16]. Overexpression of macrophages accompanied by phagocytosis of biomaterial particles further increases the local release of pro-inflammatory cytokines.

In this study, we analyzed the integration of PPE implants* in vivo* by comparing parameters of angiogenesis and inflammation in uncoated implants to implants coated with extracellular matrix components (ECM, Matrigel^®^) and implants coated with ECM and Etanercept. To allow for repeated *in vivo* analyses, we used the murine dorsal skinfold chamber model for our experiments.

## Materials and methods

### Animals and surgeries

**Fig. 1:**
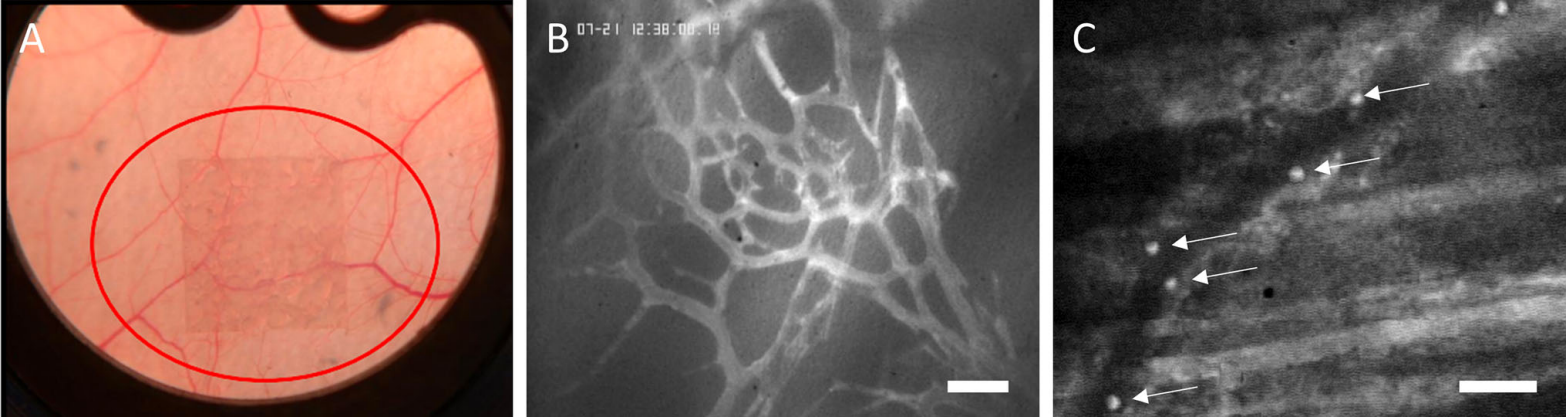
**A** Photographic image of a porous polyethylene implant (3 × 3 × 0.25 mm^3^, marked by red circle) implanted into striated muscle tissue in a dorsal skinfold chamber. **B** Exemplary image of the fluorescently labeled vessel network within the PPE implant on day 14 after biomaterial implantation. FITC dextran labeling allowed for precise identification of pre-existing and newly formed vasculature. Scale bar: 100 μm. **C** Exemplary image of Rhodamine 6G labeled leukocytes within a blood vessel in the implant material (white arrows pointing to labeled cells). Video capturing facilitated the differentiation between rolling and endothelial wall-adherent leukocytes. Scale bar: 100 μm

18 male C57BL/6 mice (Charles River, Sulzfeld, Germany) served as experimental animals for this study (*n* = 6 per experimental group). All experimental procedures performed were in accordance with institutional and governmental guidelines (Regierung von Oberbayern, Munich, Germany). All surgical procedures were performed under anesthesia with ketamine (100 mg/kg Ketavet^®^, Parke-Davis, Berlin, Germany) and xylazine (15 mg/kg Rompun^®^, Bayer, Leverkusen, Germany). The dorsal skinfold chamber surgical procedure has been previously described in detail [17, 18]. In brief, under anesthesia, the extended dorsal skin of the mouse was surgically clamped in a double layer between two symmetrical titanium frames after hair removal. On one side, a circular area of 15 mm diameter consisting of skin, subcutaneous tissue and striated skin muscle was removed. The contralateral muscle was covered with a sterile, removable glass coverslip fitted into a titanium frame. After a 48 h recovery period, the surgical field was microscopically inspected to ensure intact microcirculation and absence of inflammation. The polyethylene sample was then placed centrally into the chamber and onto the striated muscle after removal of the coverslip which was then replaced (Fig. 1A).

### Porous polyethylene implants

Porous polyethylene sheets (PPE; Medpor^®^; Stryker, Kalamazoo, MI, USA; formerly Porex Surgical, Newnan, GA; pore size 100–250 μm; thickness 250 μm) were cut into rectangular 3.0 × 3.0 mm squares and steam-sterilized. Prior to implantation, PPE implants were either bathed in sterile 0.9% saline solution or coated according to the respective experimental group. One group was coated with 50 μl growth factor reduced (GFR) BD Matrigel Matrix (Becton Dickinson, Heidelberg, Germany) or GFR BD Matrigel Matrix supplemented with 5 μl Etanercept (Enbrel^®^ at 25 mg/ml concentration, Wyeth-Ayerst Pharmaceutica Inc., PA, USA). Liquid GFR BD Matrigel entered the scaffold at 37 °C (animal body temperature).

### *In vivo* fluorescence microscopy

Experimental animals were repeatedly immobilized in a perspex tube on a custom-made stage (Effenberger, Munich, Germany) for intravital microscopy at three time points after PPE implantation (i.e. days 3, 7 and 14, see Supplemental Fig. 1). The mice remained awake during intravital microscopy and were constantly monitored for signs of discomfort or distress. A modified Zeiss microscope (Axiotech Vario; Zeiss, Göttingen, Germany) was used for imaging. Fluorescein isothiocyanate (FITC)-labeled dextran (Sigma, Deisenhofen, Germany; MW 500,000; 0.05–0.1 mL of a 5% solution in 0.9% saline) was used to stain blood plasma for the visualization of the vascular network and rhodamine 6G (Molecular Probes, Eugene, OR; 0.04 mL of a 0.05% solution in 0.9% saline) was used to stain leukocytes for the analysis of leukocyte-endothelial cell interactions (Fig. 1B, C). The fluorescent dyes were administered via tail vein injection. For selective observation of FITC-labeled plasma and rhodamine 6G-labeled leukocytes epi-illumination with a 100 W mercury lamp with selective filter blocks (Zeiss, Göttingen, Germany) was used. Both renally eliminated fluorescent markers are well-established for* in vivo* labelling and feature a blood half-life sufficient for *in-vivo* imaging over a time period of at least 2 h [19, 20].

### Microcirculatory analysis

On day 3 after biomaterial implantation, six regions of interest (ROI) per animal were randomly selected, three in the center of the porous polyethylene implant and three in adjacent connective tissue. The same ROIs were sought out again using a custom-made x-y-micrometer-stage (Effenberger, Munich, Germany) and analyzed on days 7 and 14. *In vivo* fluorescence microscopic images were acquired by a CD camera (Sony XC-77 CE; Sony, Cologne, Germany) and recorded on digital tapes (Sony DVCAM DSV 45P; Sony, Cologne, Germany) for subsequent off-line analysis. Parameters for angiogenic activity. i.e. functional vessel density in cm/cm^2^ (fvd), red blood cell velocities in mm/s (vRBC), and vessel diameters in μm (d) were measured using a specific software (Cap Image; Zeintl, Heidelberg, Germany, Supplemental Fig. 2) as previously described [21]. White blood cells were analyzed regarding the leukocyte flux in n/s, which was quantified by counting the number of cells crossing a predefined line in one vessel in 30 s. Rolling and adherent leukocytes were differentiated: rolling was defined as at least 50% of red blood cell velocity in the same vessel, adherent leukocytes were stationary for at least 30 s/mm^2^ of vessel wall surface.

## Results

### Inflammatory response

**Fig. 2:**
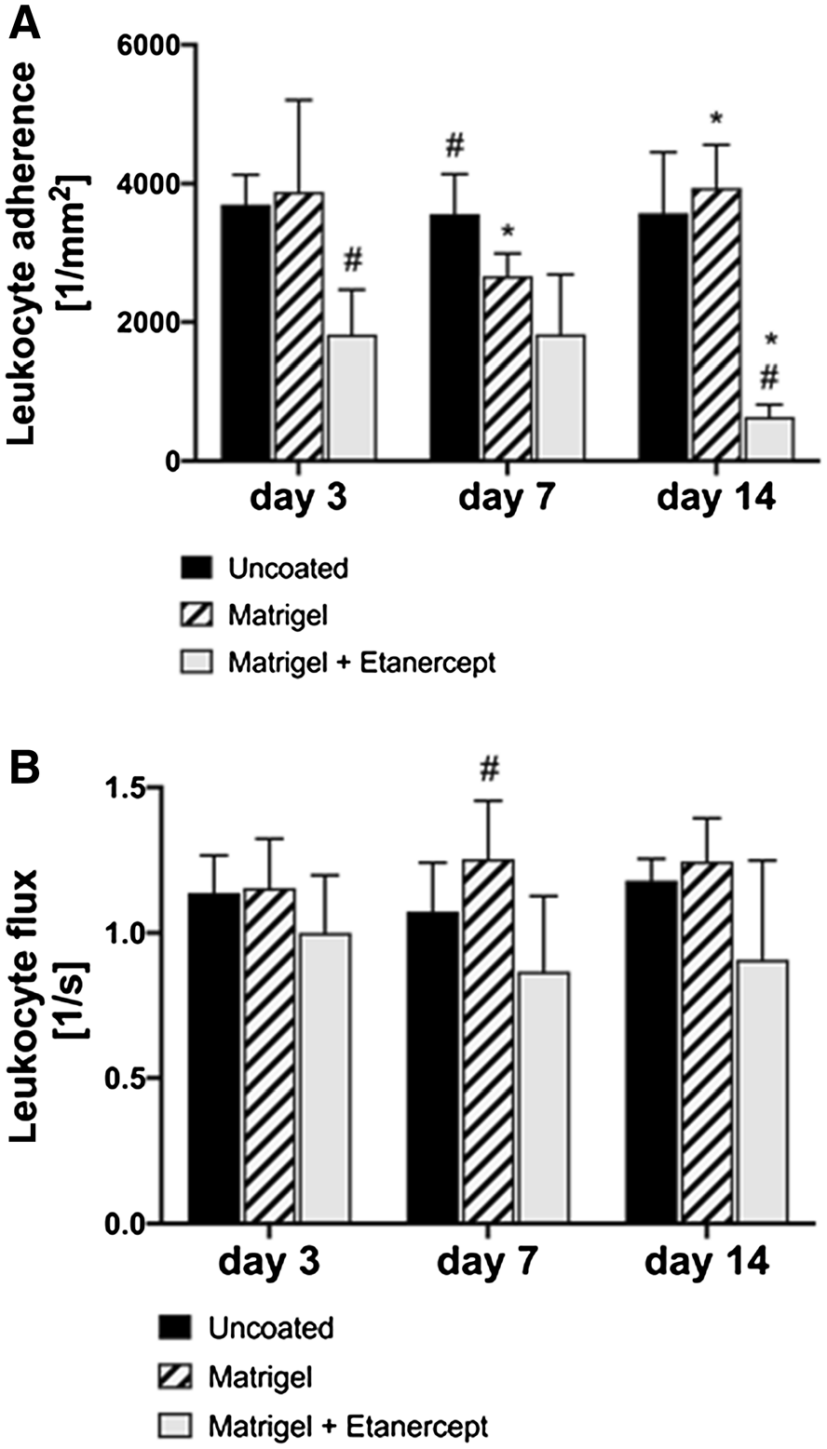
**A** On day 3, values for leukocyte-endothelial-cell adherence values (in mm^−2^) were significantly reduced in the Matrigel and Etanercept group compared to Matrigel only and uncoated implants on intra-group comparison (indicated by the following symbol: #). On day 7, both groups with coated implants showed significantly lower values compared to the uncoated implants and a significant reduction compared to the previous time point was observed for the Matrigel only group on inter-group comparison (indicated by the following symbol: *). On day 14, values for the Matrigel only group increased back to day 3 levels while the Matrigel and Etanercept group further decreased. Levels were also significantly lowered compared to both other groups on intra-group comparison. **B** Leukocyte flux values in n/s did not differ between groups and time points except for a slight increase for Matrigel-coated implants on compared to the other groups on day 7

Analysis of the initial inflammatory response showed significant differences between the three experimental groups: A reduced number of leukocytes adherent to the endothelial wall was observed for implants coated with Matrigel and Etanercept compared to both other experimental groups on day 3 (uncoated: 3699.03 mm^−2^ ± 429.27 mm^−2^ vs. Matrigel only: 3882.26 mm^−2^ ± 1318.97 mm^−2^ vs. Matrigel and Etanercept: 1821.09 mm^−2^ + 645.95 mm^−2^) and day 14 (uncoated: 3573.88 mm^−2^ ± 880.16 mm^−2^ vs. Matrigel only: 3939.09 mm^−2^ ± 623.34 mm^−2^ vs. Matrigel and Etanercept: 637.98 mm^−2^ + 176.85 mm^−2^). Implants coated with Matrigel and Etanercept were the only group to show a reduction in leukocyte-endothelial cell interactions on day 14 compared to the previous time point (Fig. 2A). Leukocyte flux values did not significantly differ between groups and time points except for a slight increase in the Matrigel group on day 7 compared the other experimental groups (Fig. 2B).

### Angiogenesis and microhemodynamics

**Fig. 3:**
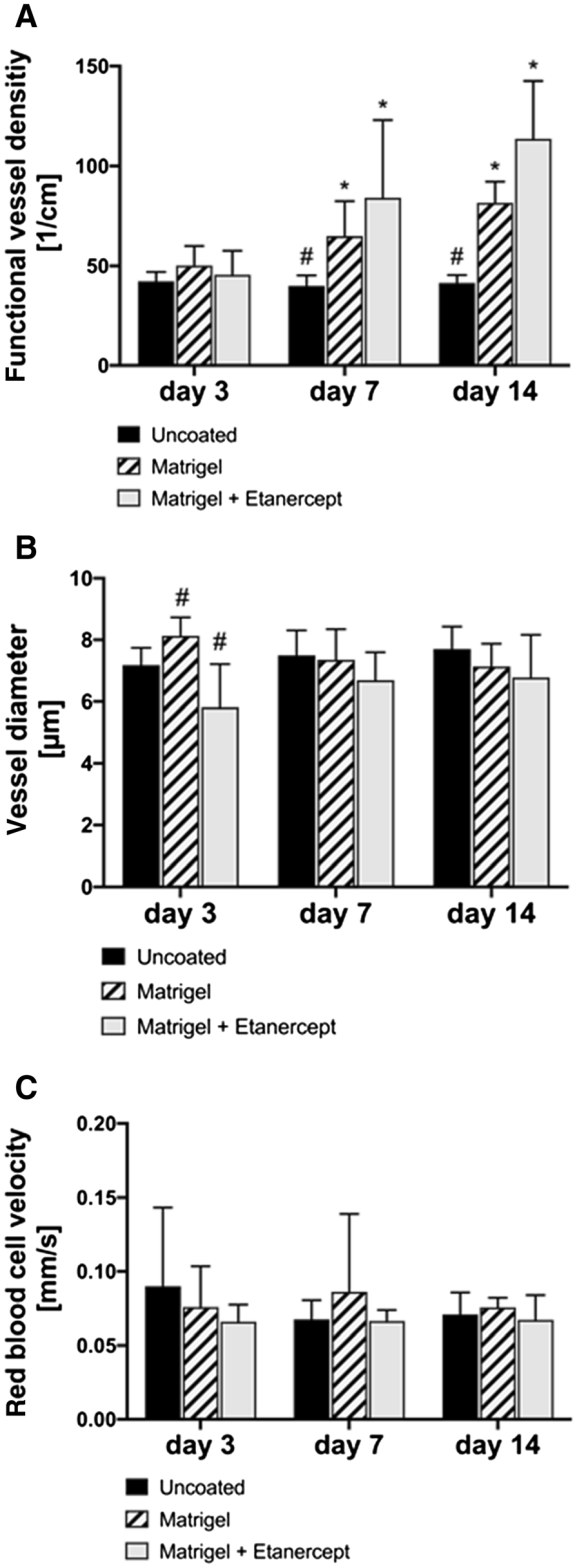
**A** Functional vessel densities (in 1/cm) did not differ on day 3. On days 7 and 14, coated implants showed an increase on the intra-group level compared to the previous time point, as well as compared to the uncoated implants on inter-group comparison at the respective time points. **B** Vessel diameters did not differ between groups or time points. **C** Red blood cell velocities did not differ between groups or time points

Quantitative analysis of microhemodynamics was also performed on days 3, 7, and 14 after implantation of PPE implants by* in vivo* fluorescence microscopy. Functional vessel densities were on the same level for all groups on day 3 (uncoated: 42.23 ± 4.76 cm/cm^2^ vs. Matrigel only: 50.20 ± 9.83 cm/cm^2^ vs. Matrigel and Etanercept: 45.53 ± 12.09 cm/cm^2^) and significantly increased for the coated implants on days 7 (uncoated: 39.94 ± 5.28 cm/cm^2^ vs. Matrigel only: 64.89 ± 17.48 cm/cm^2^ vs. Matrigel and Etanercept: 84.07 ± 38.98 cm/cm^2^) and 14 (uncoated: 41.44 ± 3.98 cm/cm^2^ vs. Matrigel only: 81.49 ± 10.65 cm/cm^2^ vs. Matrigel and Etanercept: 113.64 ± 29.00 cm/cm^2^) as shown in Fig. 3A. Differences were significant both between time points, as well as compared to uncoated implants on days 7 and 14.

Vessel diameters differed slightly, yet significantly, on day 3 on inter-group comparison but did not show any differences on days 7 and 14 (Fig. 3B). Red blood cell velocities did not differ between groups or time points (Fig. 3C).

### Mechanical integration

Implants from all three experimental groups were well integrated into the host tissue 14 days after implantation. The force necessary to dynamically dislocate the implants out of the implant bed did not differ between the three groups.

### Statistical analysis

Results are presented as mean ± SEM. Calculations were performed using the Friedman repeated measures ANOVA on ranks and Kruskal-Wallis ANOVA on ranks, respectively (SigmaStat; Jandel Scientific, San Rafael, CA). *P*-values <0.05 were considered significant.

## Discussion

A range of studies on PPE implants have shown encouraging results in improving biocompatibility and biomaterial integration by varying tissue engineering approaches [7–11, 21]. Keeping in mind that PPE is already a well-established biomaterial in reconstructive surgery with overall favorable complication rates, the necessity to further improve biocompatibility is limited to selected cases; typically, when the recipient tissue has been compromised by prior treatment. To nevertheless enable a rapid translation into clinical practice, we investigated the effects of a local treatment with TNFα receptor inhibitor Etanercept which is an anti-inflammatory agent approved for clinical use. Results were promising: Our findings suggest a reduced local inflammatory response as shown by leukocyte-endothelial cell adherence values which were significantly lower in the experimental group with ECM and Etanercept coating compared to uncoated implants or implants coated with Matrigel only. Adherent leukocytes are known to transmigrate to the interstitial space through endothelial cell tight junctions [22], hereby promoting the local inflammatory response. As shown by others, infiltrating leukocytes, primarily monocytes/macrophages, can establish foreign body giant cells which compromise the implant healing process by phagocytosis as well as the release of inflammatory cytokines [16, 23]. The pathway of increased levels of leukocyte-endothelial cell adherence via TNFα-stimulation is well established. E-selectin, P-selectin and intercellular adhesion molecule 1 (ICAM-1) are among the adhesion molecules induced by TNFα, as well as vascular cell adhesion molecule 1 (VCAM-1). Consequently, as shown by others, in TNFα-receptor- deficient mice, leukocyte-endothelial cell interactions are significantly reduced [24]. Our results showing a reduction of leukocyte-endothelial cell interactions after treatment with local Etanercept compared to both other experimental groups therefore suggest a measurable effect of local TNFα inhibition on the inflammatory response. Remarkably, the effect seems to be sustainable over a relevant period of time. In our study, leukocyte-endothelial cell interactions continuously decreased after Etanercept treatment until the final analysis time point at 14 days after implantation while levels remained roughly the same for implants from both other groups. The reported half-life of Etanercept, as reported by the manufacturer is 102 ± 30 h. The sustained effect may be attributable to the dose administered locally. Compared to the body weight-adapted weekly dose administered systemically in human patients, we chose a local one-time dose which was approximately fivefold increased. Whether lower doses may suffice to achieve a similar effect remains to be investigated follow-up dosing-range studies. Encouragingly, TNFα-inhibition did not negatively impact blood vessel ingrowth into the implant over the observation period. On the contrary, implants coated with ECM or with ECM and Etanercept both showed a significant increase in vessel density on days 7 and 14 compared to the baseline measurements on day 3 after implantation, as well as a more rapid increase in vessel density compared to the implant group of uncoated controls. This finding is reassuring because a range of anti-inflammatory agents, such as non-steroidal anti-inflammatory drugs and corticosteroids have been shown to negatively impact wound healing in the early phase [25, 26]. Our findings support the concept that selective TNFα inhibition with Etanercept does not negatively influence the healing and implant integration process, which is in line with previous findings in studies concerning bone and tendon injury analyzing breaking forces time-dependantly [27]. Similarly, we also assessed the mechanical integration of PPE implants via the assessment of dynamic breaking strengths and no differences were detected between the groups after 14 days. Interestingly, our results do not only refute any negative impact on angiogenesis by coating with Matrigel and Etanercept but in fact suggest that coating implants may promote a more rapid ingrowth of vessels in the early phase after implantation compared to uncoated implants. Vascular ingrowth is critical for successful biomaterial integration since nutrition of cells within the implant via diffusion is limited to 150–200 μm [28, 29], and any pro-angiogenic effects are supportive. TNFα-inhibition is not commonly associated with pro-angiogenic effects; in fact, in certain environments, TNFα-stimulation has been shown to promote angiogenesis via vascular endothelial growth factor, in particular [30, 31]. While we used Matrigel which is known for its pro-angiogenic potential as a carrier for Etanercept which is currently available for systemic application in humans, it is important to keep in mind that we used a growth-factor reduced version of Matrigel. Hereby we attempted to selectively analyze the effects of Etanercept and minimize the effects of additional confounding growth factors; we can therefore only hypothesize why vessel ingrowth was accelerated compared to uncoated implants. For one, even growth-factor reduced Matrigel contains a low dose of growth factors such as vascular endothelial growth factor insulin-like growth factor I (IGF-1), and epidermal growth factor (EGF), which promote tissue vascularization [32]. In addition, the pre-operative addition of Matrigel, which turns into a gel at 37 °C and is absorbed by the pores of the implant material potentially provides a carrier medium which improves the delivery of nutrients and growth factors. However, while encouraging, previous studies suggest that these angiogenesis-promoting effects of ECM components alone may be temporary and restricted to the early phase [7, 33]. Therefore, in order to reach a conclusive verdict on the pro-angiogenic effects and their impact on long-term biomaterial integration of this particular tissue engineering approach, a longer observation period would be necessary and will be addressed in future studies.

In summary, our results show a measurable, and lasting anti-inflammatory effect in PPE implants by local TNFα inhibition with Etanercept which does not compromise the angiogenic processes necessary for successful biomaterial integration. This tissue engineering approach is based on a biomaterial and a drug which are approved for clinical use in humans, which lowers the obstacles for a translation into clinical practice. Our experimental approach allowed for detailed* in vivo* measurements of inflammatory and microcirculatory parameters but is inherently limited to the early post-operative observation period. An additional long-term experimental assessment, preferably in a compromised host environment, i.e. after revision surgery, or radiation, would be desirable prior to in-patient studies and we are currently designing an appropriate follow-up study.

## Supplementary information

Below is the link to the electronic supplementary material

Timeline to illustrate the experimental process. After implantation of PPE implants into dorsal skinfold chambers of mice, intravital microscopic analysis of parameters of microcirculation and inflammation were performed at three time points, i.e. days 3, 7 and 14 after implant placement 2 (tiff 905 Kb)

(**A**)Exemplary image of the process to analyze vessel density within a specific region of interest in the implant material, as well as vessel diameter in (**B**). Vessels were marked by the researchers and respective values were computer calculated 1 (tiff 2237 Kb)
