# Supplementary figures and images for: TNF-α-Inhibition Improves the Biocompatibility of Porous Polyethylene Implants In Vivo
Source: Tissue Eng Regen Med. 2021 Jan 30;18(2):297–303. doi: 10.1007/s13770-020-00325-w (PMC8012447; doi:10.1007/s13770-020-00325-w)

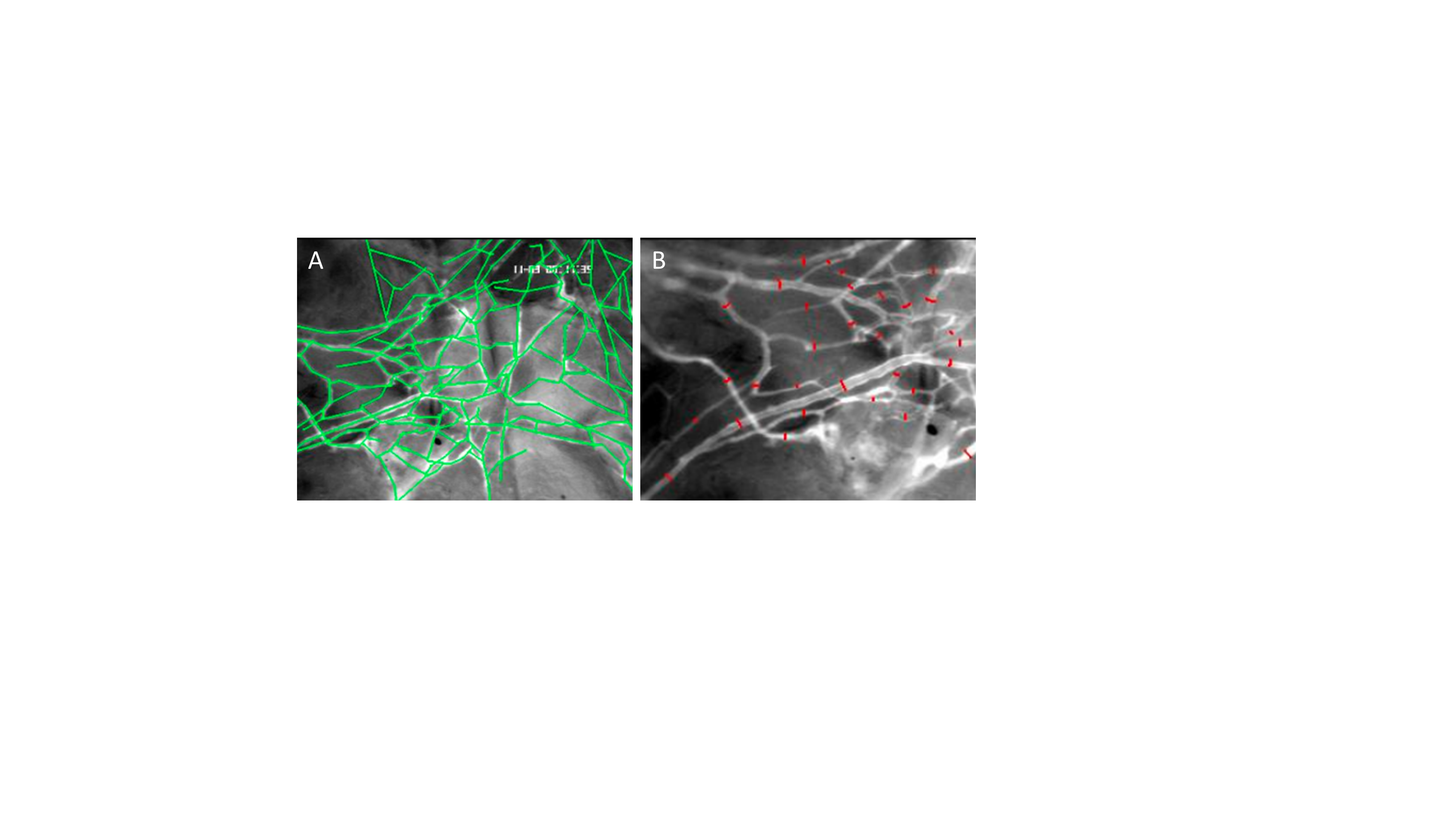

Supplement: Supplementary file 1 — Timeline to illustrate the experimental process. After implantation of PPE implants into dorsal skinfold chambers of mice, intravital microscopic analysis of parameters of microcirculation and inflammation were performed at three time points, i.e. days 3, 7 and 14 after implant placement 2 (tiff 905 Kb) [file 13770_2020_325_MOESM1_ESM.tif]

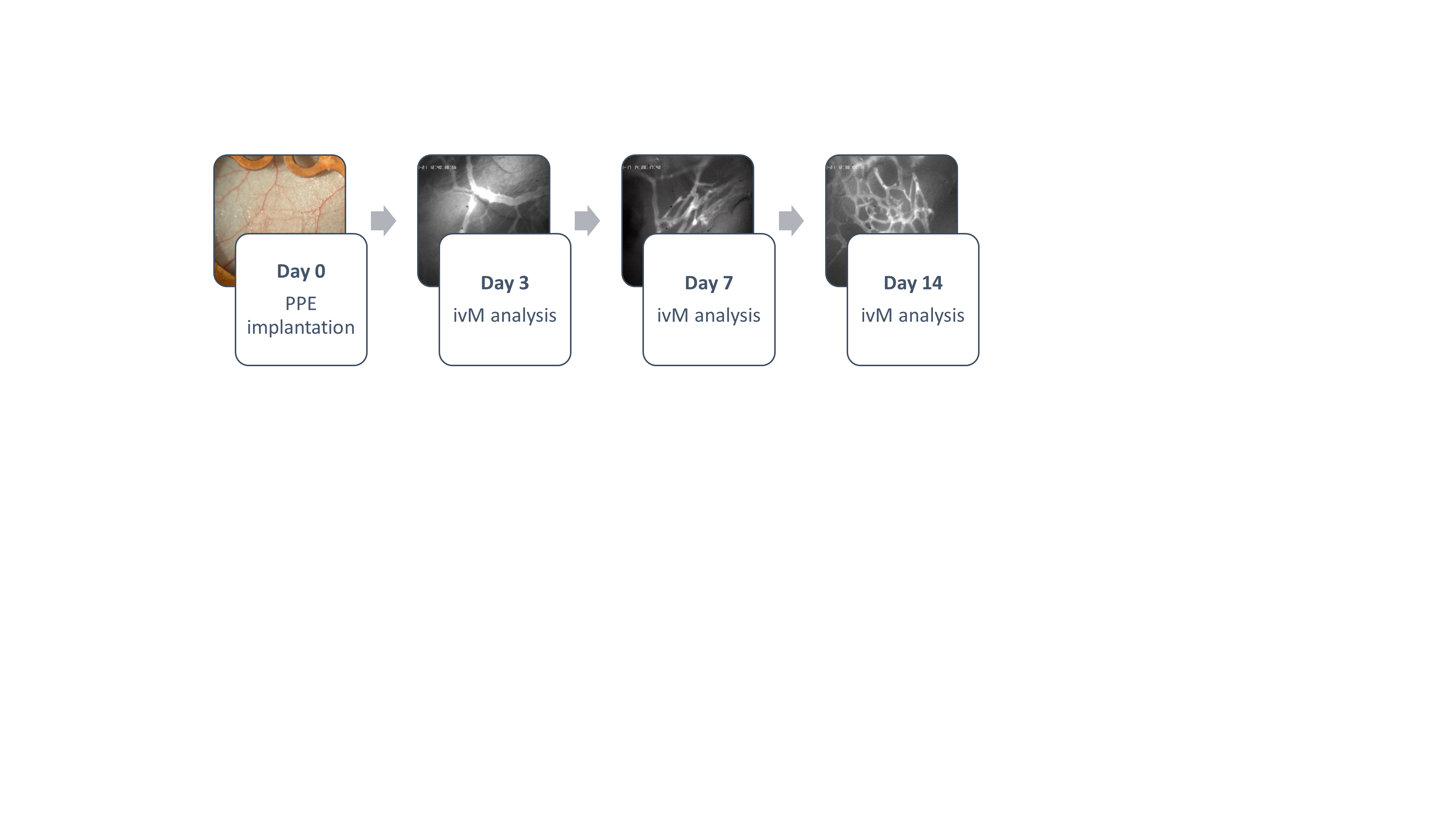

Supplement: Supplementary file 2 — (A)Exemplary image of the process to analyze vessel density within a specific region of interest in the implant material, as well as vessel diameter in (B). Vessels were marked by the researchers and respective values were computer calculated 1 (tiff 2237 Kb) [file 13770_2020_325_MOESM2_ESM.tif]
